# Postharvest Treatments with Three Yeast Strains and Their Combinations to Control *Botrytis cinerea* of Snap Beans

**DOI:** 10.3390/foods10112736

**Published:** 2021-11-09

**Authors:** Mingfang Feng, You Lv, Tiantian Li, Xinmao Li, Jiayin Liu, Xiuling Chen, Yao Zhang, Xu Chen, Aoxue Wang

**Affiliations:** 1 College of Life Sciences, Northeast Agricultural University, Harbin 150030, China; mingfangfeng@126.com (M.F.); zy13263696020@163.com (Y.Z.); 2 College of Horticulture and Landscape Architecture, Northeast Agricultural University, Harbin 150030, China; lvyou0929@163.com (Y.L.); litiantian080821@163.com (T.L.); lixinmao2020@163.com (X.L.); chenx@neau.edu.cn (X.C.); cxsteam@163.com (X.C.); 3 College of Sciences, Northeast Agricultural University, Harbin 150030, China; t18686735458@126.com

**Keywords:** snap beans, *Botrytis cinerea*, combinations, biocontrol yeasts, postharvest treatments

## Abstract

Three yeast strains, namely *Cryptococcus albidus* (Ca63), *Cryptococcus albidus* (Ca64), and *Candida parapsilosis* (Yett1006), and their combinations, including single yeast agent, two combined yeast strains, single yeast agent + NaHCO_3_, single yeast agent + chitosan, single yeast agent + ascorbic acid, and single yeast agent + konjac powder, were evaluated for their activity against *Botrytis cinerea*, the most economically important fungal pathogens causing postharvest disease of snap beans. In in vitro tests, no inhibition zone was observed in dual cultures of three yeast strains and *B. cinerea.* The mycelial growth inhibition rates of *B. cinerea* for Ca63, Ca64, and Yett1006 were 97%, 95%, and 97%, respectively. In in vivo tests, the optimal combination of the lowest disease index of snap beans with *B. cinerea* was Ca63 + Ca64, with a preventing effect of 75%. The decay rate and rust spots index of Ca64 + ascorbic acid combination were 25% and 20%, respectively, which were the lowest. The activities of defense-related enzymes increased, while malondialdehyde (MDA) content was suppressed in snap beans after different treatments. Our results highlight the potential of the three yeast strains and their combinations as new nonpolluting agents for the integrated control of *B. cinerea* on snap beans.

## 1. Introduction

Snap beans (*Phaseolus vulgaris* L. var. *chinensis* Hort.) are soft-pod variants of common beans (*Phaseolus vulgaris* L.) [1]. These are mainly eaten by pods and have good taste and high nutritional value. Due to strict requirements of geographical environment and climatic conditions for the growth of snap beans, certain good-quality snap beans are only suitable for growth in the cold regions of China, such as Heilongjiang, Jilin, and the northeast region of Inner Mongolia [1].

Unfortunately, snap beans are extremely perishable. The production loss of fruits and vegetables in industrialized and developing countries is up to 25% and 50%, respectively, during the postharvest stage due to fungal infection [2]. Diseases that cause field and postharvest losses of snap beans include common bean rust, wilt, anthracnose, botrytis, bacterial blight, and bacterial halo [3,4]. Gray mold, caused by *Botrytis cinerea*, is a well-known disease that causes severe yield losses of snap beans worldwide [5,6]. Fungal spores usually occur on the surface of fruits, and in the process of postharvest treatment the pods of snap beans can provide a suitable environment for spore germination (mainly damaged fruits) [7]. In addition, an infection may occur during storage, sales, or even after customers purchase them [6]. Because of its wide host range and huge economic losses, extensive studies have been carried out to control *B. cinerea* effectively [8]. Although fungicides have greatly reduced the incidence of gray mold, they have several disadvantages, such as environmental pollution, frequently occurring fungicide resistance, and chemical residues in food [9,10]. Therefore, there is a requirement to develop safer and more environmentally friendly disease control alternatives [11]. An alternative method is the biological control that uses biological processes to decrease the inoculation density of pathogens to limit the disease, thereby reducing the crop losses [12,13].

Recently, the use of antagonistic microorganisms to control postharvest diseases has been widely implemented. Antagonists that inhibit postharvest diseases in nature are widely used. They have many sources, including leaves, fruits, soils, and existing antagonists (including bacteria, fungi, and actinomycetes). Biocontrol yeasts use two mechanisms to inhibit the growth of target pathogens. Direct inhibition involves the secretion of antimicrobial substances, the formation of biofilms on the inner surface of wounds, and parasitic effects on pathogens. As reported, in the *Saccharomyces cerevisiae* flor strain, biofilm cells colonized the inner surface of apple wounds more effectively than planktonic cells; thereby, the development of blue mold caused by *Penicillium expansum* was controlled [14]. Indirect inhibition includes competition with pathogens for nutrient and growth space and induction of host resistance [15,16]. For example, the *Metschnikowia pulcherrima* mutant (not pigment) exhibits reduced antifungal activity [17]. The iron deficiency by fungal pathogens was believed to be one of the mechanisms by which the yeast antagonizes phytopathogenic fungi [17].

In recent years, combining biological and safe chemical substances such as food additives to prevent and control plant diseases has effectively controlled diseases and reduced chemical fungicides [18,19,20,21]. A large number of studies have shown that the combination of biocontrol agents (yeast, *Pseudomonas syringae*, *Pantoea agglomerans*, *Bacillus subtilis*, etc.) and food additives (such as chitosan, D-2-deoxyglucose, sodium bicarbonate, ascorbic acid, konjac powder, etc.) can improve the control effect of postharvest diseases of citrus, apple, lemon, tomato, carrot, pepper, cucumber, pomegranate, strawberry, cherry, kiwi fruit, loquat and other fruits and vegetables [20,22,23,24,25,26]. NaHCO_3_ is a commonly used food additive, and it is also one of the permitted ingredients in international organic food. The enhancement of the synergistic inhibitory effect of NaHCO_3_ on biocontrol agents is related to its direct inhibitory effect on pathogens. Earlier studies showed that NaHCO_3_ can inhibit the germination of pathogenic spores and the elongation of germ tubes [27]. The addition of NaHCO_3_ changes the pH and other conditions at the fruit wound and pathogens are more sensitive to these changes than biocontrol agents, so their competitiveness with biocontrol agents is weakened. This, in turn, enhances the antagonistic ability of biocontrol agents [26]. Many postharvest diseases are caused by wound pathogens, and the complete control of these diseases is achieved by using a rapidly growing and environmentally friendly agent. One of these strategies relies on the application of edible films and coatings as food-quality preservers using biopolymers (e.g., chitosan) [23,28]. Chitosan is tasteless, nontoxic, harmless, and slightly viscous, and can easily form a uniform and continuous film. It has a good preservation effect on fruits and can improve the storage quality of fruits and vegetables. Chitosan can quickly solidify into a film after being adsorbed on the surface of an object. It does not fall off or stick and has a slight permeability. Applying it to the surface of the harvested vegetables can restrict the entry of abundant O_2_ in the air and prevent the emission of CO_2_. Therefore, the respiration of the fruit is reduced; the production of other harmful substances is slowed down. The senescence of tissue cells is delayed to a certain extent, and the storage quality of fruits and vegetables is improved [18]. Secondly, the protective layer formed by chitosan on the surface of fruits and vegetables can effectively prevent the invasion of pathogens and reduce the disease infection rate in fruits and vegetables [18]. Ascorbic acid (VC) is an important water-soluble antioxidant and one of the most common food additives and endogenous substances. It can act as a scavenger of reactive oxygen species (ROS), thereby potentially protecting cells from the harmful effects of oxidation products [21]. Konjac powder has many advantages, such as high viscosity, strong water absorption, and good film-forming properties, and is often used as the active ingredient of preservatives. The antibacterial film formed by konjac powder has a barrier effect, which can delay the entry of O_2_ into fruits and vegetables and reduce the respiratory intensity, and it can be used as a carrier of antiseptic and antibacterial agents to achieve antiseptic and fresh-keeping effects [19].

In the present study, three biocontrol yeasts (Ca63, Ca64, and Yett 1006) were used. The biocontrol mechanism of these strains in controlling *B. cinerea* in the postharvest stage of snap beans was studied by in vivo and in vitro experiments. The effects of three yeast single-agent, two combinations, and fresh-keeping additives (chitosan, sodium bicarbonate, ascorbic acid, and konjac powder) in preserving snap beans were compared. The results provide a theoretical basis for the application of three biocontrol yeasts for the postharvest preservation of snap beans in a better way.

## 2. Materials and Methods

### 2.1. Plant Materials

The physiologically mature fruit of test snap beans “Zihuajiayoudou” was harvested from an experimental farm in Harbin, Heilongjiang Province, China, in August 2018. These snap beans were put in a fresh-keeping box and immediately transported back to the laboratory in a car. The snap beans selected for the test were without any physical injury or infection and had uniform size and color, and firm texture. The selected snap beans were soaked in 1000 ppm sodium hypochlorite for 1 min for disinfection, washed with tap water, and subsequently air-dried at room temperature.

### 2.2. Microorganisms and Media

The pathogen, *B. cinerea*, was isolated from infected snap beans, confirmed by the 5.8S ribosomal DNA (rDNA)-ITS and 18S rDNA sequences, and conserved in our laboratory [29].

Three strains of yeast *Cryptococcus albidus* (Ca63), *Cryptococcus albidus* (Ca64), and *Candida parapsilosis* (Yett 1006) are preserved by China General Microbiological Culture Collection Center (CGMCC). The CGMCC numbers of Ca63, Ca64 and Yett 1006 are 18,014, 1976, 18,013, respectively. Ca63, Ca64, and Yett 1006 were named by us after we isolated these strains. The numbers 18,014, 1976, 18,013 were given after identification by CGMCC. These strains were cultured on potato dextrose agar (PDA) plates. Single colony biomass was subjected to in vitro biocontrol assay or inoculated into yeast peptone dextrose (YPD) liquid media (Glucose 20 g, protein 20 g, yeast extract 10 g, distilled water 1000 mL) and incubated at 28 °C for 24 h on a rotary shaker at 200 rpm (constant temperature shaker, LKYC-1102C, Ningbo Life Technology Co., Ltd., Ningbo, China). The cell pellets were centrifuged at 8000× *g* for 10 min at 4 °C (64R, Beckman Coulter, lnc., Shanghai, China). Subsequently, the cells were resuspended in distilled water, recentrifuged, and washed twice to remove the residual culture media thoroughly. The yeast cells were resuspended in sterilized distilled water, diluted to a concentration of 10^8^ CFU mL^−1^ and used as an inoculum in in vivo assays.

### 2.3. Evaluation of Postharvest Biocontrol Ability of Three Yeast Strains and Their Combinations against Botrytis cinerea in Snap Beans

#### 2.3.1. In Vitro Biocontrol Assay

The three biocontrol yeasts were inoculated in 50 mL of yeast extract peptone dextrose (YPD) media (inoculation concentration was 2% (*v/v*)), and cultured on a rotary shaker at 200 rpm for 36 h at 28 °C. The cell pellets were obtained by centrifugation at 8000× *g* for 10 min at 4 °C. Next, the yeast cells were resuspended in sterile physiological saline (0.9%), recentrifuged, and washed twice to remove the residual culture media thoroughly. The yeast cells were resuspended in sterile physiological saline (0.9%), diluted to a concentration of 10^8^ CFU mL^−1^, and used as an inoculum in in vitro assays.

*B. cinerea*, confirmed and conserved in our laboratory, was cultured on potato dextrose agar (PDA) medium at 26 °C for 10 days. Next, 2 mL of sterile water was added to the Petri dish, the cultured spores and cells were gently scraped with a sterile blade, the cells were rinsed with sterile water, shaken well, and the hyphae were filtered with four layers of gauze to obtain pathogen spores. Spore concentrations were determined using a hemocytometer (79 mm × 39 mm × 13 mm, Shanghai Qiujing Biochemical Reagent Instrument Co., Ltd., Shanghai, China) and adjusted to 1 × 10^6^ spores per mL with sterile water.

Filter paper method [30]: on the PDA Petri dish, equidistant from the midpoint, a 5 mm diameter *B. cinerea* plug was placed on one side and a piece of filter paper with a diameter of 1 cm was placed on the other side. The distance between *B. cinerea* plug and filter paper was 20 mm. Next, 15 µL of 3 yeast suspensions at a concentration of 10^8^ CFU mL^−1^ was added dropwise to the filter paper separately. Dual cultures were incubated at 25 °C for 7 days. A Petri dish inoculated with *B. cinerea* and sterile water was used as a control, and each test was repeated three times. At the end of the incubation period, the growth of *B. cinerea* was observed.

Plate coating method [31]: the three biocontrol yeast suspensions (100 µL, in 0.9% sterile physiological saline) at a concentration of 10^8^ CFU mL^−1^ were added into a PDA solid media Petri dish and uniformly spread with a spreader. After drying, *B. cinerea* colonies, with a diameter of 5 mm, were inoculated on the media and cultured at 25 °C for 7 days. A PDA solid media Petri dish covered with sterile water and inoculated with *B. cinerea* was used as a control. The growth of *B. cinerea* was observed, and each group was treated thrice.

*B. cinerea*, with a diameter of 5 mm, was inoculated into 250 mL conical flasks containing 50 mL of potato dextrose broth (PDB) media. Next, the 3 biocontrol yeast suspensions were separately inoculated with *B. cinerea* at a concentration of 2% (*v/v*). The concentrations of the 3 yeast strains were 10^2^, 10^4^, 10^6^ and 10^8^ CFU mL^−1^, respectively. The inoculated flasks were incubated on a mini-roundabout shaker at 200 rpm at 25 °C for 7 days. The culture medium without the yeast inoculation was used as a control, and the hyphal growth of *B. cinerea* was recorded. The hyphae were filtered through gauze, dried, and weighed. The degree of growth inhibition of *B. cinerea* using the three strains was determined by the difference in dry weight of hyphae. Each treated group included three repetitions.

#### 2.3.2. In Vivo Biocontrol Assay

##### Antagonistic Activity of Three Yeasts and Their Combinations against *Botrytis cinerea*

In order to assess the efficacy of three yeasts as biocontrol agents, the method described by Czarnecka et al. [13] and Kharchoufi et al. [9], with modifications, was used. Artificial wounds were created using a sterile needle to make 5 mm deep and 5 mm wide wounds on the pods of snap beans (2 wounds per fruit). The wounds were inoculated with a single yeast agent, 2 combined yeast agents, single yeast agent + 0.2% (*m/v*) NaHCO_3_ (Tianjin Yongda Chemical Reagent Co., Ltd., Tianjin, China), single yeast agent + 0.5% (*m/v*) chitosan (Bozhou Baofeng Biological Technology Co., Ltd., Bozhou, China), single yeast agent + 1% (*m/v*) ascorbic acid (Tianjin Komiou Chemical Reagent Co., Ltd., Tianjin, China), and single yeast agent + 0.1% (*m/v*) konjac powder (Bozhou Baofeng Biological Technology Co., Ltd., Bozhou, China), wherein the yeast concentration was 10^8^ CFU mL^−1^ and the volume was 10 µL. The treated snap beans were naturally dried for 12 h, then divided into 2 groups: one group was inoculated with *B. cinerea* with a spore concentration of 1 × 10^6^ spores per mL and a volume of 10 µL; the other group was not inoculated with *B. cinerea* [32]. Then these snap beans were placed into plastic bags containing 90% relative humidity (RH) at room temperature 25 °C for 12 d. Snap beans treated with two wounds and sterilized water were used as the control group. The disease index was measured on the 12th day of storage. The other incidence of pods was recorded from the next day and counted every 3 days. The number of snap beans used in each treatment was 20, and each treatment was repeated 3 times.

Snap beans disease level [32,33]:

Level 0, the fruit does not develop.
Level 1, the disease area is less than 10% of the fruit length.
Level 2, the disease area accounts for 10% to 30% of the fruit length.
Level 3, the disease area is greater than 30% of the fruit length.

(1) $$\mathrm{Disease}\;\mathrm{index}=\frac{\Sigma \left(\mathrm{number}\;\mathrm{of}\;\mathrm{diseased}\;\mathrm{fruits}\;\mathrm{per}\;\mathrm{level}\times \mathrm{corresponding}\;\mathrm{disease}\;\mathrm{level}\right)}{\mathrm{total}\;\mathrm{number}\;\mathrm{of}\;\mathrm{investigations}\times \mathrm{maximum}\;\mathrm{disease}\;\mathrm{level}}\times 100$$

(2) $$\mathrm{Control}\;\mathrm{effect}=\frac{\mathrm{disease}\;\mathrm{index}\;\mathrm{of}\;\mathrm{control}\;\mathrm{group}-\mathrm{disease}\;\mathrm{index}\;\mathrm{of}\;\mathrm{treated}\;\mathrm{group}}{\mathrm{disease}\;\mathrm{index}\;\mathrm{of}\;\mathrm{control}\;\mathrm{group}}\times 100\%$$

##### The Growth Dynamics of Three Yeasts on the Surface of Snap Beans

Snap beans of same size, no disease, and no mechanical damage were selected and processed as described in Section 2.1. The 3 kinds of biocontrol yeast suspensions (100 µL, 10^8^ CFU mL^−1^, in 0.9% sterile NaCl solution) were evenly sprayed on the surface of the pods. Then these snap beans were placed into plastic bags containing 90% RH at room temperature for 96 h. The number of yeasts was determined every 12 h. The method for determining the number of yeasts cells on the surface of the pods was as follows: The processed pods were soaked in 500 mL sterile water, cleaned ultrasonically for 5 min, and then the number of yeasts in the cleaning solution was detected by the plate coating method using a microscope [34]. The test was repeated three times.

##### Storage Tests of Postharvest Snap Beans

Snap beans with same size, no disease, and no mechanical damage were selected. The postharvest fruit storage test was conducted after processing as described in Section 2.1, following the method of Wang et al. [35] with slight modifications. Single yeast agent, two combined yeast agents, single yeast agent + 0.2% (*m/v*) NaHCO3, single yeast agent + 0.5% (*m/v*) chitosan, single yeast agent + 1% (*m/v*) ascorbic acid, and single yeast agent + 0.1% (*m/v*) konjac powder with a concentration of 10^8^ CFU mL^−1^ and a volume of 3 mL were sprayed evenly on snap beans. The amount of snap beans used in each treatment was 300 ± 5 g. The snap beans with different treatments were dried naturally at room temperature. Then these were placed into plastic bags containing 90% RH at room temperature. Snap beans treated with sterile water were used as the control group. The decay period of the snap beans in different treated groups was observed every 3 days for 12 days. The other quality indexes of the fruits were determined every 3 days for 12 days. Each treatment was repeated thrice.

(1) Mass loss: Mass loss of snap beans was detected by the weighing method with balance instruments. The mass loss of the snap beans was measured from the beginning of storage and measured every 3 days [32,35].

The following formula was used to calculate the mass loss:

(3) $$\mathrm{Weight}\;\mathrm{loss}\;\mathrm{rate}=\frac{\mathrm{Pre-storage}\;\mathrm{weight}-\mathrm{weight}\;\mathrm{after}\;\mathrm{storage}}{\mathrm{Pre-storage}\;\mathrm{weight}}\times 100\%$$

(2) Rate of decay: The size of the rot area on snap beans was divided into four levels:

Level 0, no decay.
Level 1, the decayed area is less than 20% of the whole area.
Level 2, the decayed area accounts for 20% to 50% of the whole area.
Level 3, the decayed area accounts for 50% to 70% of the whole area.

The following formula was used to calculate the decay index:

(4) $$\mathrm{Decay}\;\mathrm{rate}=\frac{\Sigma \left(\mathrm{decay}\;\mathrm{level}\times \mathrm{number}\;\mathrm{of}\;\mathrm{pods}\;\mathrm{at}\;\mathrm{this}\;\mathrm{level}\right)}{\mathrm{highest}\;\mathrm{rot}\;\mathrm{level}\times \mathrm{total}\;\mathrm{number}\;\mathrm{of}\;\mathrm{pods}}\times 100\%$$

(3) Rust spots are a common phenomenon in the storage of snap beans, related to cold damage, relative humidity in the storage environment, and gas composition and concentration [36]. The smaller the rust spots index, the lighter is the occurrence of rust spots. The rust spots index was divided into four levels according to the degree of rust occurrence on the surface of snap beans [36].

Level 0, no rust spots.
Level 1, less rust with commodity value.
Level 2, more rust spots, no commodity value.
Level 3, serious rust spots, loss of food quality.

(5) $$\mathrm{Rust}\;\mathrm{spots}\;\mathrm{index}=\frac{\Sigma \left(\mathrm{rust}\;\mathrm{spots}\;\mathrm{level}\times \mathrm{number}\;\mathrm{of}\;\mathrm{samples}\right)}{\mathrm{highest}\;\mathrm{level}\times \mathrm{total}\;\mathrm{number}\;\mathrm{of}\;\mathrm{samples}}\times 100\;$$

(4) Ascorbic acid (Vitamin C, VC) content

The VC content was determined according to trade standards of agricultural products of the People’s Republic of China: GB5009.86–2016, with modifications. The specific steps are as follows:

The sample of snap beans was appropriately weighed, and a small amount of 1% oxalic acid was then added, ground into slurry with a mortar, and filtered with a gauze. The filtrate was transferred to a 50 mL volumetric flask and diluted with 2% oxalic acid. Next, 1 mL of standard ascorbic acid solution (0.1 mg mL^−1^) was taken, 9 mL of oxalic acid solution (1%, *v/v*) was added and titrated with 0.1% 2, 6-dichlorophenol indole in a micro-burette. The endpoint of titration was that the light red color did not fade within 15 s. The T value (average value) was calculated from the volume of the dye used. Next, the amount of VC (mg, fresh weight) equivalent to 1 mL of dye was calculated. Two more samples were treated in the same manner.

(6) $$m=\frac{\mathrm{VT}}{m_{0}}\times 100$$

where

m: 100 g sample contains the mass of VC (g).
V: dye volume used in titration (L).
T: mass of VC per milligram of dye can be oxidized (g L^−1^).
m_0_: 10 mL sample containing sample mass (kg).

(5) Chlorophyll content and soluble protein content

Snap beans (0.5 g) were collected and cut into pieces. Distilled water (1 mL) and a small amount of calcium carbonate were added for grinding. Then, 10 mL of extract solution (absolute ethanol:acetone = 1:2 (*v/v)*) was used and extracted for 3 h in the dark. The chlorophyll content (fresh weight) was determined according to the instructions provided in the kit (Beijing Solarbio Science & Technology Co., Ltd., Beijing, China) once the residue turned white [37].

Snap beans (0.5 g) were collected, and physiological saline was added to them according to the weight (g):volume (mL) = 1:9 to prepare a 10% tissue homogenate in an ice-water bath. The supernatant was collected after centrifugation at 1000× *g* for 10 min and mixed with physiological saline at a ratio of 1:4 (*v/v*). The soluble protein content was measured according to the instructions provided in the kit (Nanjing Jiancheng Technology Co., Ltd., Nanjing, China) [37].

##### Assay of Defense-Related Mechanisms

The content of malondialdehyde (MDA) was detected by the thiobarbituric acid (TBA) method. Snap beans (0.5 g) were collected, and 0.9% NaCl solution was added according to the weight (g):volume (mL) = 1:9 to prepare a 10% tissue homogenate in an ice-water bath. The protein concentration (Cp) of the 10% tissue homogenate was measured. Then, we operated according to the Table 1.

We covered the centrifuge tubes and tied a small hole with a needle on each cover. We mixed the tubes with a vortex mixer. After having them in a 95 °C water bath for 40 min, we took them out and cooled them with running water. Then, they were centrifuged at 2000× *g* for 10 min. The supernatant was used to measure the optical density (OD) value at 532 nm. For the preparation method of reagents I, II and III, see the kit manual (Nanjing Jiancheng Technology Co., Ltd., Nanjing, China).

The calculation formula of MDA content in the sample is as follows:

(7) $$\mathrm{MDA}\;\mathrm{content}\;=\frac{\mathrm{ODt}-\mathrm{ODc}}{\mathrm{ODs}-\mathrm{ODb}}\times \mathrm{standard}\;\mathrm{concentration}÷\mathrm{Cp}\;(\mathrm{mol}\;{\mathrm{kg}}^{-1})\;$$

where

ODt: OD value of test tube.
ODc: OD value of control tube.
ODs: OD value of standard tube.
ODb: OD value of blank tube.
Cp: protein concentration (mg mL^−1^).
Standard concentration: 10 nmol mL^−1^.

Catalase (CAT) activity was determined by the ammonium molybdate method. Snap beans (0.5 g) were collected and 0.9% NaCl solution was added according to the weight (g):volume (mL) = 1:9 to prepare a 10% tissue homogenate in an ice-water bath. The 10% tissue homogenate was centrifuged at 1000× *g* for 10 min. We took the supernatant, added 0.9% NaCl solution at 1:4 to dilute it, and determined the protein concentration (CP). Then, we operated according to the Table 2.

We mixed the supernatant well and measured the OD value of the supernatant at 405 nm. For the preparation method of reagents I, II, III and Ⅳ, see the kit manual (Nanjing Jiancheng Technology Co., Ltd., Nanjing, China).

Unit definition: the amount of 1 μmol H_2_O_2_ decomposed per second per milligram of tissue protein is a unit of activity.

The calculation formula of CAT activity in the sample is as follows:

(8) $$\mathrm{CAT}\;\mathrm{activity}=\left(\mathrm{ODc}-\mathrm{ODt}\right)\times 271\times \frac{1}{60\times 0.05}÷\mathrm{Cp}\;(U\;{\mathrm{mg}}^{-1})$$

where

ODt: OD value of test tube.
ODc: OD value of control tube.
Cp: protein concentration (mg mL^−1^).
Note: 271 is the reciprocal of the slope.

Phenylalnine ammonialyase (PAL) catalyzes the cleavage of L-phenylalanine into trans cinnamic acid and ammonia. Trans cinnamic acid has the maximum OD value at 290 nm. The PAL activity is calculated by measuring the change of OD value. Snap beans (0.5 g) were collected, and extract solution was added to weight (g):Volume (mL) = 1:9 to prepare a 10% tissue homogenate in an ice-water bath. The 10% tissue homogenate was centrifuged at 8000× *g* for 10 min. We took the supernatant, then operated according to the Table 3.

Mixed the supernatant well, stood for 10 min, and measured the OD value of the supernatant at 290 nm. For the preparation method of reagents R2, R3, R4 and crude enzyme solution, see the kit manual (Nanjing Jiancheng Technology Co., Ltd., Nanjing, China) [35].

Unit definition: 0.1 change in 290 nm OD value per minute per g of tissue in each mL reaction system is a unit of enzyme activity.

The calculation formula of PAL activity in the sample is as follows:

(9) $$\mathrm{PAL}\;\mathrm{activity}=\frac{\mathrm{ODt}-\mathrm{ODb}}{0.1}÷\left(\frac{\mathrm{sample}\;\mathrm{weight}}{\mathrm{crude}\;\mathrm{enzyme}\;\mathrm{solution}\;\mathrm{volume}}\times \mathrm{sample}\;\mathrm{volume}\right)\times \frac{\mathrm{total}\;\mathrm{volume}\;\mathrm{of}\;\mathrm{reaction}\;\mathrm{system}}{1}÷\mathrm{reaction}\;\mathrm{time}=\;16.6\times \left(\mathrm{Am}-\mathrm{Ab}\right)÷\mathrm{sample}\;\mathrm{weight}\;(U\;g^{-1})$$

where

ODt: OD value of test tube.
ODb: OD value of blank tube.

The activity of superoxide dismutase (SOD) was determined by the 4-[3-(4-iodophenyl)-2-(4-nitrophenyl)-2 H-5-tetrazolio]-1,3-benzene disulfonate (WST-1) method. Snap beans (0.5 g) were collected and 0.9% NaCl solution was added according to the weight (g):volume (mL) = 1:4 to prepare a 20% tissue homogenate in an ice-water bath. The 20% tissue homogenate was centrifuged at 2000× *g* for 10 min. We took the supernatant, then operated according to the Table 4.

The supernatant was mixed well, incubated at 37 °C for 20 min, and the absorbance value of the supernatant was determined at 450 nm by microplate reader. For the preparation method of the enzyme working solution, enzyme diluent solution and substrate application solution, see the kit manual (Nanjing Jiancheng Technology Co., Ltd., Nanjing, China) [35].

Unit definition: in this reaction system, when the SOD inhibition rate reaches 50%, the corresponding enzyme amount is a SOD activity unit (U).

The calculation formula of SOD activity in the sample is as follows:

(10) $$\mathrm{SOD}\;\mathrm{inhibition}\;\mathrm{rate}\;\left(\%\right)=\frac{\left(\mathrm{Ac}-\mathrm{Acb}\right)-\left(\mathrm{At}-\mathrm{Atb}\right)}{\mathrm{Ac}-\mathrm{Acb}}\times 100\%$$

where

At: absorbance value of test hole.
Atb: absorbance value of test blank hole.
Ac: absorbance value of control hole.
Acb: absorbance value of control blank hole.

(11) $$\mathrm{SOD}\;\mathrm{activity}=\;\mathrm{SOD}\;\mathrm{inhibition}\;\mathrm{rate}÷50\%\times \mathrm{dilution}\;\mathrm{ratio}\;\mathrm{of}\;\mathrm{reaction}\;\mathrm{system}\times \mathrm{dilution}\;\mathrm{ratio}\;\mathrm{before}\;\mathrm{test}÷\;\frac{\mathrm{sample}\;\mathrm{weight}\;\left(g\right)}{\mathrm{homogenate}\;\mathrm{buffer}\;\mathrm{added}\;\left(\mathrm{mL}\right)}\;(U\;g^{-1})$$

### 2.4. Statistical Analysis

The data obtained were subjected to a one-way analysis of variance (ANOVA) followed by Duncan’s multiple range test with statistical product and service solutions (SPSS) 20.0 software. Data are presented as mean ± SD with three replicates per treatment. Significant differences were considered at *p* < 0.05 and respective significant differences were marked using different letters.

## 3. Results

### 3.1. Evaluation of Postharvest Biocontrol Ability of Three Yeast Strains and Their Combinations against Botrytis cinerea in Snap Beans

#### 3.1.1. In Vitro Biocontrol Assay

**Figure 1:**
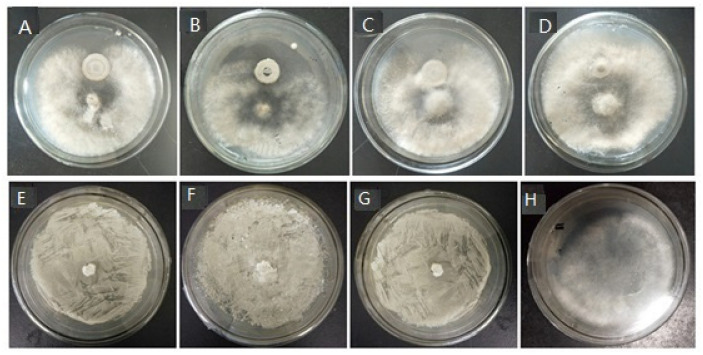
Antifungal activity of three biocontrol yeast strains against *Botrytis cinerea*. (**A**–**H**) represent Ca63, Ca64, Yett 1006, and control, respectively. The upper row shows the results of filter paper method, and the lower row shows the results of plate coating method.

Results of the filter paper method showed that the three yeast strains could not inhibit *B. cinerea.* The filter paper could not be covered by the hyphae of *B. cinerea,* indicating that these strains cannot produce effective fungal resistant substances to inhibit the pathogen (Figure 1). In contrast, *B. cinerea* growth was inhibited by the three strains in the test of plate coating method. Although a few *B. cinerea* were visible, the three strains of yeast grew well, indicating that these strains could effectively compete with the pathogen for nutrition and space to reproduce (Figure 1).

**Figure 2:**
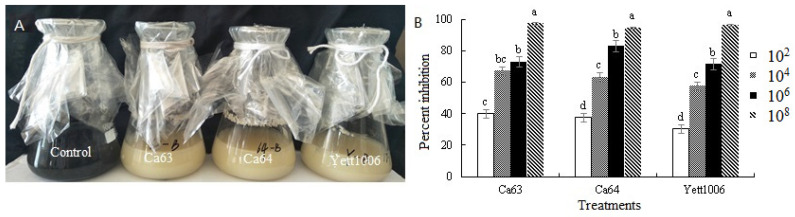
Evaluation of biocontrol abilities of three yeast strains against *Botrytis cinerea*. (**A**) Inhibition effect of three biocontrol yeast strains (10^8^ CFU mL^−1^) on mycelia growth of *Botrytis cinerea* after 7 days of incubation. (**B**) Inhibition rate of three biocontrol yeast strains against *Botrytis cinerea*. Error bars represent the standard deviations of means (*n* = 3), and different letters within the same figure stand for significant differences among four concentration levels at *p* < 0.05.

The inhibitory effect of these strains on *B. cinerea* hyphae is shown in Figure 2 after incubation of *B. cinerea* with 3 strains for 7 days at 25 °C. At a concentration of 10^8^ CFU mL^−1^ of 3 yeast suspensions, *B. cinerea* hyphae in the culture solution were mostly invisible, indicating the highest inhibition rate of hyphae occurred at this concentration. The inhibition rates of Ca63, Ca64, and Yett1006 against *B. cinerea*, calculated after the hyphae were dried and weighed, were 97%, 95%, and 97%, respectively. Moreover, the inhibition rate of *B. cinerea* reached 50% or more at a concentration of 10^4^ CFU mL^−1^ of three strain suspensions.

#### 3.1.2. In Vivo Biocontrol Assay

##### Antagonistic Activity of Three Yeast Strains and Their Combinations against *Botrytis cinerea*

**Figure 3:**
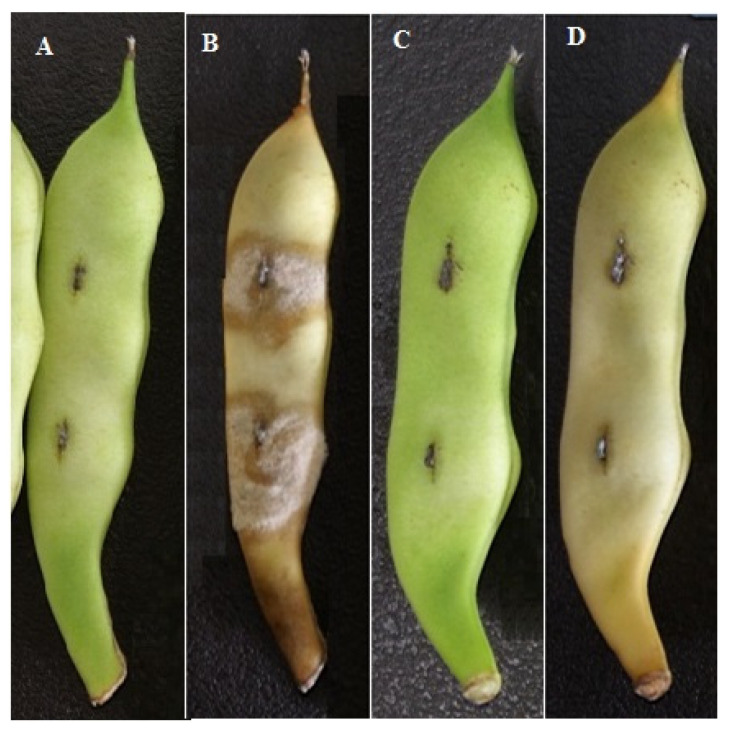
Evaluation of biocontrol abilities of three yeast strains and their combinations against *Botrytis cinerea* in vivo. (**A**) Control group: Snap beans treated with 2 wounds and sterilized water inoculated with *Botrytis cinerea* after 3 days; (**B**) Control group: Snap beans treated with 2 wounds and sterilized water inoculated with *Botrytis cinerea* after 12 days; (**C**) Treated group: Snap beans treated with 2 wounds and Ca63 + Ca64 inoculated with *Botrytis cinerea* after 3 days; (**D**) Treated group: Snap beans treated with 2 wounds and Ca63 + Ca64 inoculated with *Botrytis cinerea* after 12 days.

The disease index of three yeast strains and their combinations significantly differed among the treated groups. Table 1 and Table 2 show the biocontrol effect of three strains and their combinations on snap beans inoculated without and with *B. cinerea*, respectively. The lowest disease indices of 27 and 18 were observed in the group treated with Ca63 and Ca63 + Ca64, and the control effects were 66% and 75%, respectively (Table 5 and Table 6, Figure 3). Differences existed between the three strains with the same adjuvant.

##### The Growth Dynamics of Three Yeasts on the Surface of Snap Beans

**Figure 4:**
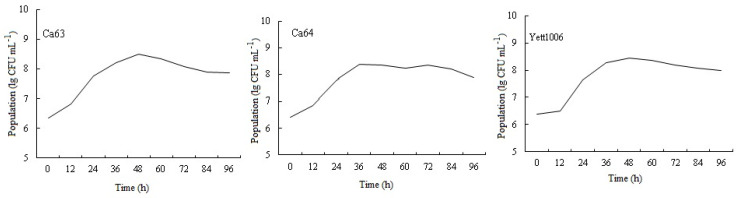
Growth dynamics of three yeast strains on the surface of snap beans.

The dynamic changes of the 3 biocontrol yeasts on the pods showed that the 3 yeasts can colonize on the surface of the snap beans and had a growth process, reaching the maximum value in 36~48 h, and the concentration can reach 3.14 × 10^8^ CFU mL^−1^, 4 × 10^8^ CFU mL^−1^, 2.8 × 10^8^ CFU mL^−1^, respectively (Figure 4). The number of the 3 yeasts’ cells decreased slightly after 48 h, but the values were relatively stable during the investigation period.

##### Storage Tests of Postharvest Snap Beans

Compared with the control group, snap beans treated with different combinations of three yeast strains had a lower decay rate and rust spots index. Moreover, there were differences between the combinations (Table 7). The decay rate and rust spots index of three yeast strains separately combined with ascorbic acid were low. For the decay rate, Ca64 + ascorbic acid, Ca63 + ascorbic acid and Ca63 + Ca64 treated group were the best. As for the rust spots index, Ca64 + ascorbic acid combination was the most effective; the decay rate and rust spots index of Ca64 + ascorbic acid-treated snap beans were 25% and 20%, respectively, significantly lower than those of the control, which accounted for 50% and 54%, respectively.

**Figure 5:**
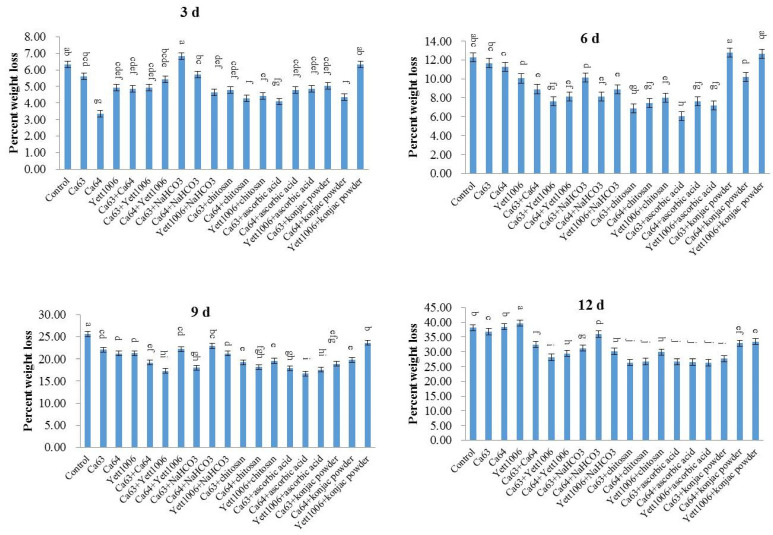
Evaluation of weight loss in snap beans between different treatments. Error bars represent the standard deviations of means (*n* = 3). On the bars, the small letters indicate a significant difference in the treatment according to Duncan’s multiple difference test (*p* < 0.05).

The main reasons for the mass loss of snap beans during storage are transpiration and respiration that result in considerable loss of fruit moisture. Figure 5 shows that the weight loss rate of Ca64 + ascorbic acid-treated snap beans was 6% on the sixth day, which was 54% of the control. On the ninth day of storage, the weight loss rates of snap beans in Ca64 + ascorbic acid and Ca63 + Yett1006-treated groups were both 17%, both of which were significantly lower than those of control. In general, each treatment reduced the moisture loss in snap beans to a varying extent, and the weight loss rate of three biocontrol yeast combined-treated groups was less than that of the three yeasts alone.

**Figure 6:**
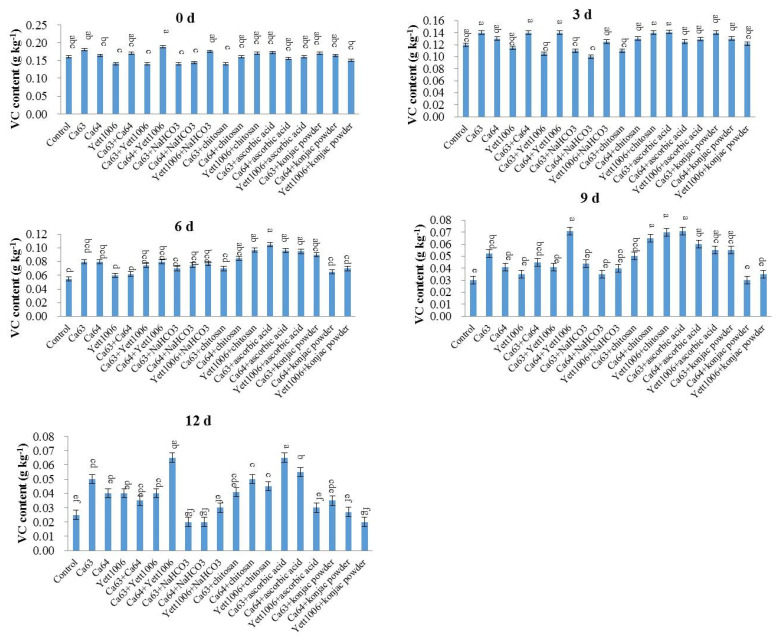
Evaluation of Vitamin C content in snap beans between different treatments. Error bars represent the standard deviations of means (*n* = 3). On the bars, the small letters indicate a significant difference in the treatment according to Duncan’s multiple difference test (*p* < 0.05).

Figure 6 shows that VC content in each treated group gradually decreased as the number of storage days increased. The VC content in each treated group was reduced in the first 3 days of storage, with no significant difference as compared with the control. The VC content of the control group from the third to sixth days decreased rapidly, whereas the decrease in the Yett1006 + chitosan-treated group was the slowest and was significantly different from that in the control. After 9 days of storage, the VC content of Yett1006 + chitosan- and Ca64 + ascorbic acid-treated group was the lowest as compared with the initial content, which was significantly different from that of the control, thus delaying the loss of VC during the storage period.

**Figure 7:**
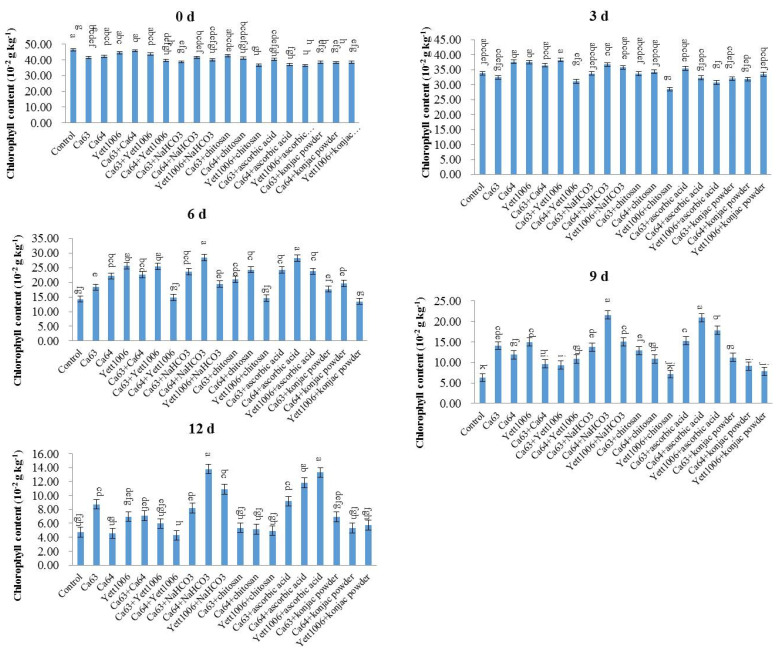
Evaluation of chlorophyll content in snap beans between different treatments. Error bars represent the standard deviations of means (*n* = 3). On the bars, the small letters indicate a significant difference in the treatment according to Duncan’s multiple difference test (*p* < 0.05).

The chlorophyll content of each treated group during storage of snap beans is shown in Figure 7. The chlorophyll content reduced during the storage period, and the rate of decline increased after the third day of storage. The decline rate of chlorophyll content in each treated group was lower than that in the control in the first 3 to 6 days, indicating that each treated group could alleviate the yellowing of pods to a different extent. The combination with the highest chlorophyll level was Ca64 + ascorbic acid, with its chlorophyll content only 31% lower than the initial content, which was significantly different from that of the control.

**Figure 8:**
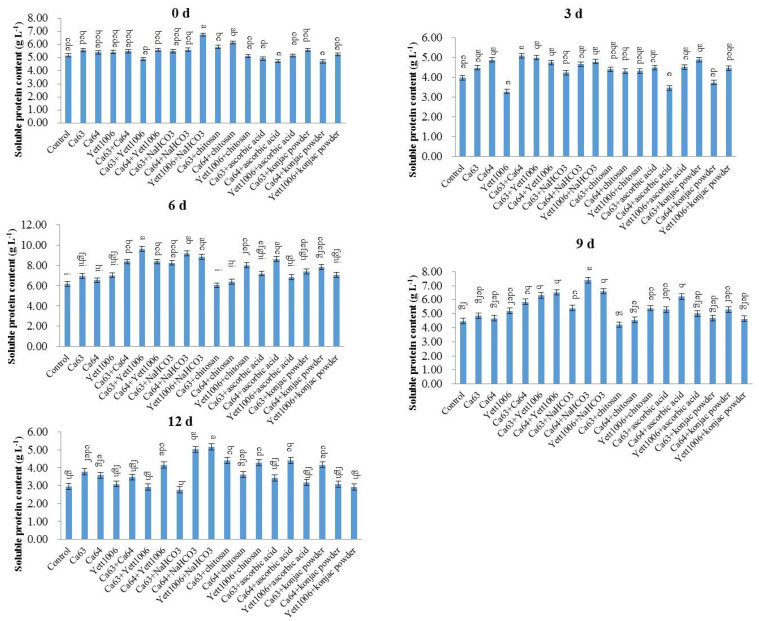
Evaluation of soluble protein content in snap beans between different treatments. Error bars represent the standard deviations of means (*n* = 3). On the bars, the small letters indicate a significant difference in the treatment according to Duncan’s multiple difference test (*p* < 0.05).

As shown in Figure 8, the soluble protein content of snap beans treated with different combinations decreased slightly in the early storage period, followed by an increase in the middle of the storage and a rapid decrease at the end of storage. On the ninth day of storage, the lowest decrease in the soluble protein content (22%) was reported in the Ca64 + chitosan-treated group. The increase in the soluble protein content during storage could be related to the change in the activity of defense enzymes during storage. The soluble protein content at the end of storage decreased rapidly due to the decrease in the degradation activity of defense enzymes during the aging of the fruit tissue. The rapid decrease in the soluble protein content indicated a decline in the quality of snap beans, with a gradual loss of storage value.

##### Assay of Defense-Related Mechanisms

(1) Evaluation of MDA content in snap beans between different treatments

**Figure 9:**
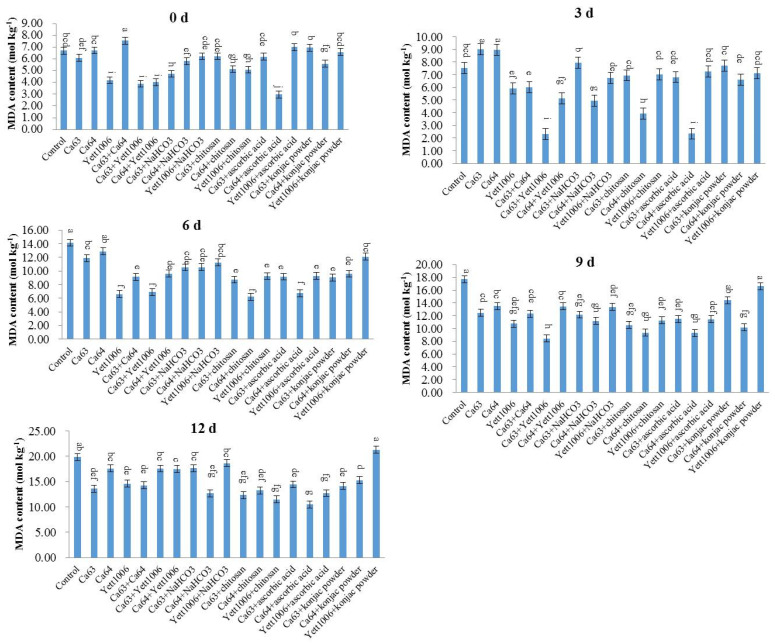
Evaluation of MDA content in snap beans between different treatments. Error bars represent the standard deviations of means (*n* = 3). On the bars, the small letters indicate a significant difference in the treatment according to Duncan’s multiple difference test (*p* < 0.05).

MDA is a product of cell membranes or lipidation. Under stress or during aging, the generation of free radicals and their scavenging mechanisms in plants decline. Free radicals can damage cell membranes, gradually converting them into MDA. Therefore, the MDA content can reflect the degree of senescence of plants. As shown in Figure 9, the change in the MDA content in the treated groups in the early stage of storage was not considerably different. On the ninth day after storage, the MDA content of each treated group was lower than that of the control, with the content of MDA in the Ca64 + ascorbic acid-treated group the lowest.

(2) Evaluation of SOD activity in snap beans between different treatments

**Figure 10:**
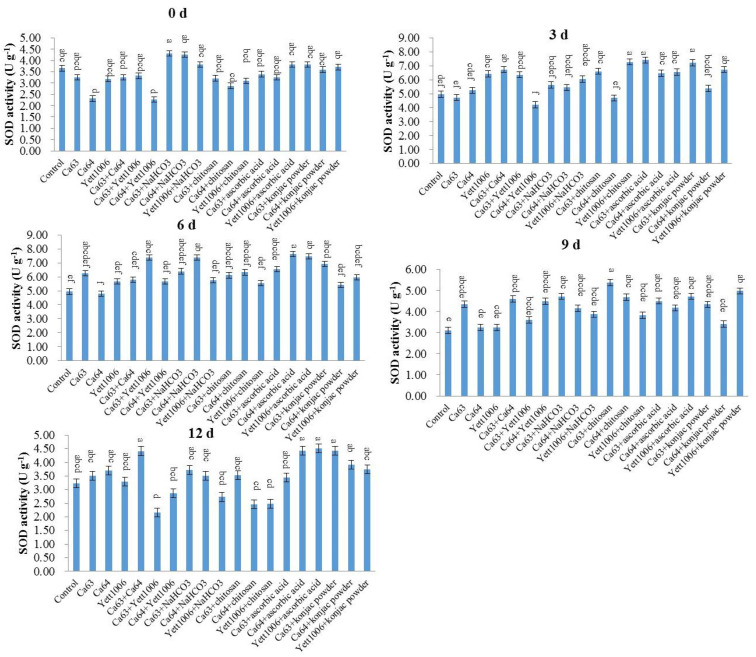
Evaluation of SOD activity in snap beans between different treatments. Error bars represent the standard deviations of means (*n* = 3). On the bars, the small letters indicate a significant difference in the treatment according to Duncan’s multiple difference test (*p* < 0.05).

Figure 10 shows that as the storage time prolonged, the SOD activity first increased and then decreased. The SOD activity of each treated group improved in the early stage of storage, with the SOD activity of the Ca63 + Ca64-treated group reaching the maximum on the third day, which was 2.21 times the initial value. The SOD activity of each treated group on the sixth day of storage was higher than that of the control, indicating that this treatment promoted the SOD activity during storage. However, the SOD activity of the control group increased slightly at the end of storage, which could be related to the infection by the pathogen at the end of the storage period.

(3) Evaluation of PAL activity in snap beans between different treatments

**Figure 11:**
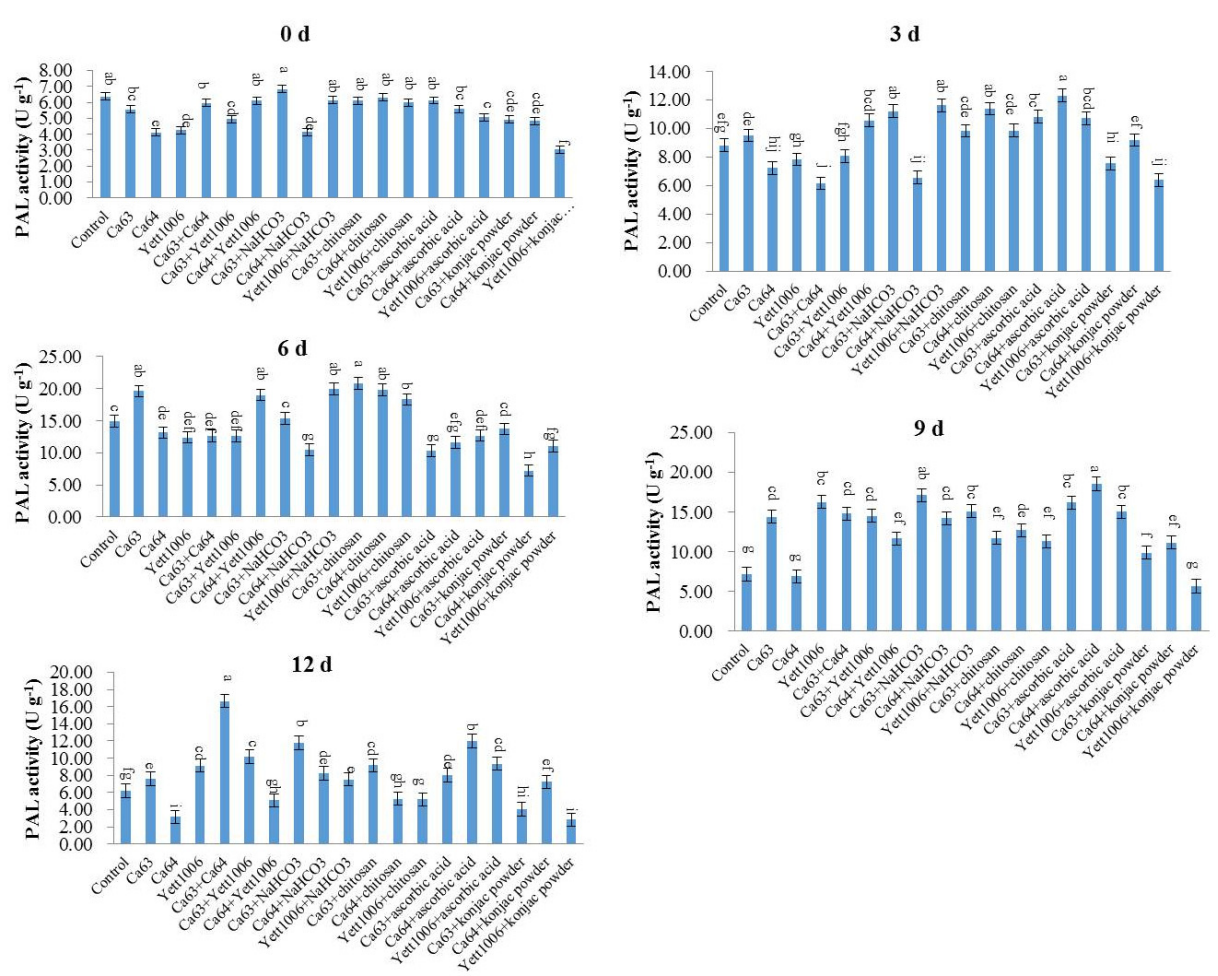
Evaluation of PAL activity in snap beans between different treatments. Error bars represent the standard deviations of means (*n* = 3). On the bars, the small letters indicate a significant difference in the treatment according to Duncan’s multiple difference test (*p* < 0.05).

Figure 11 shows that the PAL activity of snap beans increased in each treated group during the early stage of storage, primarily related to fruit resistance development by three biocontrol yeasts. The PAL activity of three biocontrol yeast combination-treated groups was higher than that of three yeast single-treated groups. The PAL activity of the Ca63 + Ca64-treated group was 1.79 times the initial value, whereas that of the control group increased only slightly during the storage. In addition, Ca64 + ascorbic acid-treated snap beans still maintained a high PAL activity at the end of the storage, which was significantly different from that of the control.

(4) Evaluation of CAT activity in snap beans between different treatments

**Figure 12:**
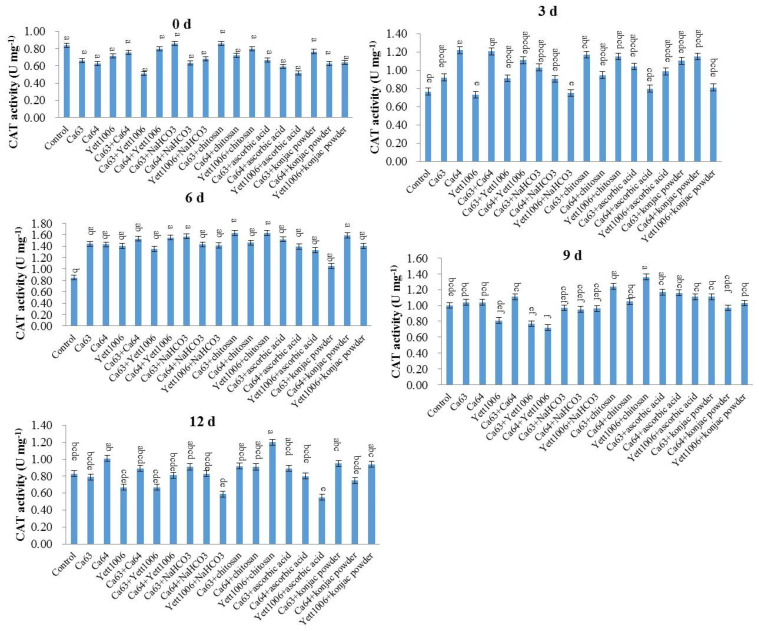
Evaluation of CAT activity in snap beans between different treatments. Error bars represent the standard deviations of means (*n* = 3). On the bars, the small letters indicate a significant difference in the treatment according to Duncan’s multiple difference test (*p* < 0.05).

The effect of different combinations of yeast strains on the CAT activity of snap beans during storage is shown in Figure 12. During the first 6 days of storage, the CAT activity of each treated group increased continuously and reached a peak on the sixth day. The CAT activity in the control group reached a peak on the ninth day, indicating that different treatments promoted the CAT activity of snap beans to different degrees in the early stage of storage. The Yett1006 + ascorbic acid-treated group maintained a high CAT activity during the storage period, and the CAT activity on the sixth day was 2.74 times the original value, whereas that of the control was only 1.08 times of the initial value.

## 4. Discussion

### 4.1. Evaluation of Biocontrol Ability of Three Yeast Strains and Their Combinations against Botrytis cinerea in Snap Beans

Based on the results of in vivo and in vitro experiments, we believe that the three biocontrol yeast strains inhibited the growth of the pathogen on the pods primarily by two mechanisms. First, these strains had good adhesion to the surface of the fruit and the wound. The biocontrol ability of antagonistic microorganisms is related to their ability to colonize on the surface or the wound of the fruit [11,38,39]. The dynamic changes in three biocontrol yeasts on the pods in this study indicated (Figure 4) that these could colonize well on the surface of snap beans and multiply rapidly in a short time. Moreover, the number of yeast cells was stable at the later stage of the investigation, which was related to their better adaptability to the environment and utilization of nutrients on the surface of snap beans. Second, the three yeast strains can compete with pathogens for nutrition and space through rapid reproduction. Yeast preferentially grows, thereby making the nutrition and space unavailable to the pathogen, thereby limiting its growth. Similar results of other antagonist yeasts have been reported [40,41,42,43,44,45,46].

### 4.2. Evaluation of Storage Quality of Snap Beans between Different Combinations of Three Yeast Strains

The formation of biofilms can also be considered as a specific and very successful strategy to compete for space [14]. Biofilms are microbial communities that live and grow on a surface and consist of a single species or represent a multispecies alliance [47]. Compared with free-floating cells, biofilms may exhibit very different properties and are considered to be virulence factors for pathogenic microorganisms [47,48]. In the biological control of yeast, the formation of biofilm, mainly in the leaf layer and pericarp layer (i.e., in the wounds), is now considered to be an important mode of action [14]. For example, the formation of biofilm in apple wounds can prevent postharvest disease [49]. In this study, the three yeasts and their different combinations can form biofilms on the surface of snap beans and block the growth and reproduction of pathogens so they can effectively prevent pathogens. Therefore, for determining the in vivo biological activity of three strains of yeast and their combinations, we first inoculated the three yeasts and their combinations on snap bean surfaces to form a biofilm, and then inoculated the pathogenic microbes.

The storage test of snap beans showed that different combinations of three yeast strains had an inhibitory effect on the postharvest diseases of snap beans. The postharvest snap beans had reduced nutritional content and quality due to respiration and transpiration during storage. The combination of yeasts with food additives in this test had different positive effects on the postharvest quality of snap beans, which significantly preserved the quality of snap beans.

It has been reported that NaHCO_3_ has a positive effect on the prevention and treatment of postharvest diseases of citrus, carrots, peppers, tomatoes, bananas and lemons [20,22,24,26]. In this study, after the three yeast strains were used in combination with NaHCO_3_, the disease index decreased significantly except for the Ca64 combination (it decreased slightly). The decay rate of all combinations decreased significantly. For the rust spots index, except for the Yett1006 combination (it increased slightly), there was no significant change.

Schena et al. [50] reported that the combined treatment of postharvest biocontrol yeast *Candida saitoana* and chitosan significantly improved the inhibition of postharvest diseases of grapes. EI-Ghaouth et al. [51] showed that postharvest biocontrol yeast *C. Saitoana* can effectively cooperate with chitosan to enhance the control of *Penicillium expansum*, *B. cinerea*, and *P. digitatum*. *Cryptococcus laurentii* was combined with water-soluble chitosan to treat postharvest *Malus micromalus* fruit. After a storage period, the test results of various indicators were significantly better than those of the yeast-alone treatment group [18]. Similarly, chitosan incorporated with tea seed oil coatings was able to prolong the shelf-life of pears, and tea seed oil could improve chitosan activity against *B. cinerea* [52]. Our study showed that after the three yeast strains were used in combination with chitosan, the disease index decreased, but not significantly. The decay rate of all combinations decreased significantly. The rust spots index of all combinations decreased significantly except for the Yett1006 combination, where it increased slightly.

Li et al. [53] pointed out that VC enhances the oxidative stress tolerance of *Pseudomonas caribbean* and has biological control effects against postharvest rot in apples. Singh et al. (2020) [54] observed that VC effectively induced the germination of seeds through seed priming and antagonistic microorganisms, and triggered the defense mechanism to control tomato plant *Fusarium* wilt. Yang et al. [21] showed that VC treatment significantly induced the metabolic pathway of *P. caribbean*, so VC potentially enhanced the biological control effect of *P. caribbean* on diseases after apple harvest. In this study, after the combination of three yeast strains and ascorbic acid, the disease index decreased significantly except for the Ca64 combination (it slightly increased). The decay rate of all combinations decreased significantly. For the rust spots index, all three combinations decreased, among which the Ca64 combination showed significant drop.

Konjac powder is used in the storage, transportation and preservation of fruits and vegetables. There are many applications for its use in tomato, cucumber, pomegranate, strawberry, cherry, kiwi fruit, loquat, etc. [25]. In our study, after the three yeast strains were used in combination with konjac powder, there was no significant change in the disease index except for a slight decrease in Ca64 combination. There was no significant change in the decay rate of all combinations. For the rust spots index, Ca63 combination was slightly higher, Ca64 combination was slightly lower, and Yett1006 combination was significantly higher.

The decay rate, rust spots index, and weight loss rate of snap beans in different treatment groups reduced as compared with the control. Moreover, reduced VC content, chlorophyll content, and soluble protein content improved as compared with control. The decay rate and rust spots index of Ca64 + ascorbic acid-treated groups were 25% and 20%, respectively, which were significantly lower than those of the control group. In addition, the VC content of this group decreased slowly from the sixth to ninth day, such that the Ca64 + ascorbic acid-treated group had the best quality of snap beans.

In summary, the overall effect of the three yeasts combined with ascorbic acid is better than the combined use with NaHCO_3_, chitosan, and konjac powder, and also better than the three yeasts alone. The optimal combination of the lowest disease index was Ca63 + Ca64, while the decay rate and rust spots index of Ca64 + ascorbic acid combination were the lowest. The mechanism of the combined application of Ca63 + Ca64 to reduce the disease index of snap beans remains to be further studied.

### 4.3. Activation of Defense Enzymes of Snap Beans by Three Biocontrol Yeasts

In response to external adverse conditions, the defense system of plants gets activated and strengthens their resistance. During a pathogenic infection and senescence in fruits and vegetables, enzyme activities are significantly altered. This affects physiological, biochemical, and metabolic pathways in plants. For example, SOD, PAL, and CAT are closely related to the development of plant disease resistance [13].

SOD can catalyze the superoxide anion radical disproportionation reaction in plants to generate O_2_ and H_2_O_2_, which reduces the toxic effects on plants. It is a major free radical-scavenging enzyme in plant cells. CAT eliminates excessive reactive oxygen species formed as part of cell aging. It alleviates the damage caused by these free radicals to plant cells and tissues, ensures metabolism balance, and delays the process of postharvest aging. PAL is an important defense enzyme that participates in plant growth, development, and aging. When plants are under biotic or abiotic stress, PAL activity increases rapidly [55,56]. We found that the activity of SOD, PAL, and CAT increased in the early stage of storage, and the content of MDA increased slowly; the change in the MDA content indicated fruit aging. The three strains of biocontrol yeasts induced stress response in snap beans upon infection and activated the defense enzymes [57,58,59]. However, the study on the role of *Debaryomyces hansenii* and *Wickerhamomyces anomalus* in the biological control of *Monilinia fructicola* in apples reported that the activity of peroxidase (POD) and CAT changed significantly. The POD activity of *Debaryomyces hansenii* and *Wickerhamomyces anomalus* (67% and 54%) was significantly higher than that of untreated control fruits, whereas the CAT activity of these (65% and 68%) was significantly lower than that of untreated control fruits, which was contrary to the results of our study (CAT activity increased) [13]. Changes in the activity of SOD, PAL, and CAT in plants changed the levels of reactive oxygen species (ROS). ROS play a dual role in the interaction between plants and pathogens. They also function as mobile signaling molecules and induce several molecular, biochemical, and physiological responses in infected plant cells [60,61].

On the one hand, biocontrol yeasts enhance hypersensitive response (HR) by activating SOD, PAL, and CAT, thus producing excessive ROS to resist pathogens. Furthermore, they may induce diffusion of oxidative burst by reducing SOD, PAL, and CAT activities, leading to the death of host cells and yeast cells at the wound site, known as positive regulatory effects of ROS. On the other hand, biocontrol yeasts can reduce ROS production by activating SOD, PAL, and CAT, thus stimulating the plant defense system against pathogens, which is a negative regulatory effect of ROS [13]. Thus, the effect of biocontrol yeasts on enzyme activity differs due to differences between their biological control activity and spectrum. This phenomenon is related to their participation in plant defense response [13]. However, the mechanism of inducing immunity by biocontrol yeasts in plants attacked by pathogens has not been clarified. Therefore, further studies are required to understand the changes in ROS in plants treated with biocontrol yeasts before and after infection to elucidate the specific regulatory mechanism.

### 4.4. Safety Evaluation of the Three Biocontrol Yeasts

*C. albidus* was used as a biocontrol agent in the product Yieldplus^®^. The product was registered in 1997 and sold by anchor Bio Technologies in South Africa [14]. Yieldplus^®^ was designed for grapefruit and citrus fruits against *B. cinerea* and *Penicillium expansum* [14]. It was later proved to effectively control *B. cinerea* during postharvest cold storage of strawberries [62]. In humans, *Candida* yeasts, mainly found in the gastrointestinal tract of healthy adults, accounts for 80% of all fungal infections in hospitals, resulting in blood, urinary tract, and surgical site infections [63]. *Candida albicans* is still considered the most common species causing candidiasis, but the incidence rate of this pathogen has decreased [64]. Currently, *Candida* non *albicans*, such as *C. parapsilosis* sensulato *Candida tropicalis*, have become the second or third major causes of *Candida* infections, which are related to a high incidence rate and mortality [64]. Although *C. parapsilosis* has pathogenic potential and developed antifungal resistance, it has been reported as a biocontrol agent. *C. parapsilosis* showed notable inhibitory activities against *Fusarium* isolates (which are active producers of secondary toxic and carcinogenic metabolites such as fumonisin) and *Aspergillus* species [65,66]. Contamination of food and feed products with fumonisin and aflatoxin can endanger the health of humans and animals, and lead to agricultural loss. Therefore, *C. parapsilosis* could be considered as a biocontrol agent against the growth and fumonisin production of toxigenic *Fusarium* species and aflatoxin production by aflatoxigenic *Aspergillus* species [65,66].

## 5. Conclusions

In conclusion, the three yeast strains, namely Ca63, Ca64, and Yett 1006, including single yeast agent, two combined yeast strains, single yeast agent + NaHCO_3_, single yeast agent + chitosan, single yeast agent + ascorbic acid, and single yeast agent + konjac powder, showed high potential as biocontrol agents to control postharvest disease of snap beans caused by *Botrytis cinerea*. The optimal combination of the lowest disease index was Ca63 + Ca64, with a preventing effect of 75%. In addition, the decay rate and rust spots index of Ca64 + ascorbic acid combination were 25% and 20%, respectively, which were the lowest. Potential action modes of three yeasts include competition for space and nutrients with pathogens; ability to activate defense-related enzymes, such as SOD, PAL, and CAT; reduce the MDA content, enhance plants’ resistance, and delay plants’ senescence.

## Figures and Tables

**Table 1:** Operation method.

Reagent Name	Blank Tube	Standard Tube	Test Tube	Control Tube
10 nmol mL^−1^ Standard solution (mL)	-	0.2	-	-
Absolute ethanol (mL)	0.2	-	-	-
Test sample (mL)	-	-	0.2	0.2
Reagent I (mL)	0.2	0.2	0.2	0.2
Mixed well (shook the centrifuge tubes a few times)				
Reagent Ⅱ (mL)	3	3	3	3
Reagent Ⅲ (mL)	1	1	1	-
50% Glacial acetic acid (mL)	-	-	-	1

**Table 2:** Operation method.

Reagent Name	Control Tube	Test Tube
Tissue homogenate (mL)	-	0.05
Reagent I (mL) (Preheating at 37 °C)	1.0	1.0
Reagent Ⅱ (mL) (Preheating at 37 °C)	0.1	0.1
Mixed well and reacted accurately at 37 °C for 1 min		
Reagent Ⅲ (mL)	1.0	1.0
Reagent Ⅳ (mL)	0.1	0.1
Tissue homogenate (mL)	0.05	-

**Table 3:** Operation method.

Reagent Name	Test Tube	Blank Tube
Crude enzyme solution (µL)	40	-
R2: Buffer solution (µL)	1480	1520
R3: Substrate solution (µL)	400	400
Mixed well, accurately water bath at 30 °C for 30 min		
R4: Termination solution (µL)	80	80

**Table 4:** Operation method.

Reagent Name	Control Hole	Control Blank Hole	Test Hole	Test Blank Hole
Test sample (µL)	-	-	20	20
Distilled water (µL)	20	20	-	-
Enzyme working solution (µL)	20	-	20	-
Enzyme diluent solution (µL)	-	20	-	20
Substrate application solution (µL)	200	200	200	200

**Table 5:** Biocontrol effect of three strains and their different combinations on snap beans not inoculated with *B. cinerea*.

Treatment	Disease Index	Control Effect (%)
Ca63	27 ± 2.08 g	66
Ca64	37 ± 1.92 ef	55
Yett1006	30 ± 2.35 fg	63
Ca63 + Ca64	41 ± 1.18 cde	51
Ca63 + Yett1006	48 ± 2.35 bc	41
Ca64 + Yett1006	52 ± 1.92 b	35
Ca63 + NaHCO_3_	43 ± 1.92 bcde	46
Ca64 + NaHCO_3_	42 ± 3.85 cde	48
Yett1006 + NaHCO_3_	38 ± 3.85 def	53
Ca63 + chitosan	48 ± 1.92 bc	41
Ca64 + chitosan	42 ± 3.85 cde	48
Yett1006 + chitosan	47 ± 1.92 bcd	42
Ca63 + ascorbic acid	43 ± 1.92 bcde	46
Ca64 + ascorbic acid	52 ± 1.92 b	35
Yett1006 + ascorbic acid	31 ± 1.92 fg	61
Ca63 + konjac powder	49 ± 1.28 bc	39
Ca64 + konjac powder	48 ± 2.80 bc	41
Yett1006 + konjac powder	47 ± 1.92 bcd	42
Control (Filtered water)	76 ± 1.97 a	-

Note: The letter after each column of data is Duncan’s multiple difference detection value at 0.05 level.

**Table 6:** Biocontrol effect of three strains and their different combinations on snap beans inoculated with *Botrytis cinerea*.

Treatment	Disease Index	Control Effect (%)
Ca63	33 ± 1.58 b	54
Ca64	25 ± 2.18 c	66
Yett1006	33 ± 1.70 b	55
Ca63 + Ca64	18 ± 1.98 d	75
Ca63 + Yett1006	24 ± 1.43c	66
Ca64 + Yett1006	32 ±1.40 bc	55
Ca63 + NaHCO_3_	24 ± 1.82 c	67
Ca64 + NaHCO_3_	21 ± 2.03 cd	71
Yett1006 + NaHCO_3_	25 ± 1.63 c	66
Ca63 + chitosan	29 ± 1.29 bc	60
Ca64 + chitosan	23 ± 1.24 c	68
Yett1006 + chitosan	31 ± 2.49 bc	57
Ca63 + ascorbic acid	20 ± 3.79 d	73
Ca64 + ascorbic acid	28 ± 2.94 cd	62
Yett1006 + ascorbic acid	25 ± 1.78 c	66
Ca63 + konjac powder	33 ± 2.14 b	54
Ca64 + konjac powder	22 ± 3.01 cd	69
Yett1006 + konjac powder	33 ± 2.08 b	55
Control (Filtered water)	72 ± 1.08 a	-

Note: The letter after each column of data is Duncan’s multiple difference detection value at 0.05 level.

**Table 7:** Evaluation of decay rate and rust spots index of snap beans following different yeast strains and yeast strains combination treatments.

Treatment	Decay Rate (%)	Rust Spots Index
Ca63	37 ± 1.39 b	27 ± 1.11 c
Ca64	37 ± 2.30 b	29 ± 2.20 b
Yett1006	36 ± 1.25 b	27 ± 1.05 c
Ca63 + Ca64	28 ± 2.36 d	29 ± 1.00 b
Ca63 + Yett1006	31 ± 2.00 cd	26 ± 1.56 cd
Ca64 + Yett1006	28 ± 2.19 cd	23 ± 1.27 d
Ca63 + NaHCO_3_	30 ± 2.20 cd	27 ± 0.61 c
Ca64 + NaHCO_3_	32 ± 3.05 c	30 ± 2.08 b
Yett1006 + NaHCO_3_	31 ± 0.89 cd	31 ± 0.99 b
Ca63 + chitosan	32 ± 2.22cd	23 ± 0.87 d
Ca64 + chitosan	27 ± 2.11 cd	26 ± 1.00 cd
Yett1006 + chitosan	29 ± 1.65 cd	28 ± 1.09 bc
Ca63 + ascorbic acid	28 ± 1.84 d	26 ± 0.49 cd
Ca64 + ascorbic acid	25 ± 0.69 d	20 ± 1.33 d
Yett1006 + ascorbic acid	28 ± 2.04 cd	26 ± 1.96 cd
Ca63 + konjac powder	39 ± 1.92 b	30 ± 1.91 bc
Ca64 + konjac powder	37 ± 2.60 b	28 ± 0.87 b
Yett1006 + konjac powder	38 ± 2.31 b	32 ± 1.89 b
Control	51 ± 3.11 a	38 ± 1.46 a

Note: The letter after each column of data is Duncan’s multiple difference detection value at 0.05 level.

## Data Availability

The data presented in this study are available on request from the corresponding author.
